# Growth hormone alleviates oxidative stress and improves oocyte quality in Chinese women with polycystic ovary syndrome: a randomized controlled trial

**DOI:** 10.1038/s41598-020-75107-4

**Published:** 2020-10-30

**Authors:** Yan Gong, Shan Luo, Ping Fan, Song Jin, Huili Zhu, Tang Deng, Yi Quan, Wei Huang

**Affiliations:** 1 Department of Obstetrics and Gynecology, West China Second University Hospital of Sichuan University, Chengdu, Sichuan People’s Republic of China; 2 Key Laboratory of Birth Defects and Related Diseases of Women and Children, Ministry of Education, Chengdu, Sichuan People’s Republic of China; 3 Reproductive Medicine Centre, Sichuan Provincial Women’s and Children’s Hospital, Chengdu, Sichuan People’s Republic of China; 4 Laboratory of Genetic Disease and Perinatal Medicine, Key Laboratory of Birth Defects and Related Diseases of Women and Children, Ministry of Education, Chengdu, Sichuan People’s Republic of China; 5 Department of Reproductive Medicine, West China Second University Hospital of Sichuan University, #1416 Chenglong Road, JinJiang District, Chengdu, 610041 Sichuan People’s Republic of China

**Keywords:** Cell biology, Drug discovery, Biomarkers, Diseases, Endocrinology, Medical research

## Abstract

Oxidative stress (OS) is associated with poor oocyte quality and in vitro fertilization and embryo transfer (IVF-ET) outcomes for patients with polycystic ovary syndrome (PCOS). Growth hormone (GH) can function to reduce OS in some types of cells. Therefore, this prospective randomized study investigated whether GH can significantly improve OS and oocyte quality in women with PCOS. This study enrolled 109 and 50 patients with and without PCOS (controls), respectively. The patients with PCOS were randomly assigned to receive treatment with GH (PCOS-T) or not (PCOS-C). The primary outcome included markers of OS in serum and FF, and secondary outcomes were mitochondrial function in granulosa cells (GCs) and IVF-ET outcomes. The PCOS groups showed higher basal serum total oxidant status (TOS) and OS index (OSI) levels. The follicle fluid (FF) TOS and OSI and GC apoptosis rate were significantly higher, whereas the GC mitochondrial membrane potential (MMP) was significantly lower in the PCOS-C group than in the PCOS-T and non-PCOS control groups (*P* < 0.05). Significantly more oocytes were fertilised and cleavage stage embryos were produced in the PCOS-T group than in the PCOS-C group (*P* < 0.05). GH also improved the rates of implantation and clinical pregnancy, but not significantly (*P* > 0.05). This study showed that GH alleviated the TOS and OSI level in FF and improved GC mitochondrial dysfunction and oocyte quality in patients with PCOS.

**Clinical Trial Registration Number:** This project was prospectively registered on the Chinese Clinical Trial Registry on October 20, 2018. (ChiCTR1800019437) (https://www.chictr.org.cn/edit.aspx?pid=28663&htm=4).

## Introduction

Polycystic ovary syndrome (PCOS) is a prevalent endocrine and metabolic disorder that affects 5–10% of women of reproductive age and has an infertility rate of 6.36%^1^. The pathophysiology of this syndrome is complicated and not well elucidated. Chronic anovulation caused by hyperandrogenaemia and/or insulin resistance (IR) results in decreased fecundity^1^. Heterogeneous endocrine dysfunctions not only arrest folliculogenesis, but also result in unsatisfactory pregnancy outcomes with higher spontaneous abortion rates^2^. The first-line therapy for infertile women with PCOS who desire to conception is ovulation induction with letrozole, clomiphene citrate, or one of them plus gonadotropin. However, in vitro fertilization (IVF) and embryo transfer (ET) were needed by women with PCOS who failed pregnancy with ovulation induction treatment, or infertile with fallopian or male factor. The available evidence remains controversial about whether women with PCOS experienced lower embryo quality and unsatisfactory pregnancy outcomes of IVF-ET despite the generation of more oocytes^2–6^. Except PCOS and its comorbidities arrest follicle development and maturation, intra-ovarian factors negatively influence granulosa cell (GC)-oocyte interaction, oocyte maturation, fertilization, and potential embryonic development competence^2,7,8^.

Low to moderate levels of reactive oxygen species (ROS) and/or reactive nitrogen species (RNS) are involved in physiological processes including defence against infections, cellular signalling systems, and cell growth and differentiation^9^. Excessive ROS and RNS can damage the innate antioxidant defence system and destroy proteins, DNA, and lipids. Oxidative stress (OS) is defined as an imbalance derived from excessive ROS/RNS production and/or decreased antioxidant defences in pathological states^10^.

The pathophysiology of PCOS, including hyperandrogenaemia, obesity, IR and anovulation, is associated with OS^11–13^. Markers of OS in serum, follicle fluid (FF) and mitochondrial dysfunction in GCs are associated with poor oocyte quality and IVF outcomes for patients with PCOS^8,9,13,14^.

Antioxidants such as vitamin E, vitamin C, omega-3 fatty acids, coenzyme Q10 (CoQ10), selenium and metformin play key roles in reducing OS levels^15–18^. However, most antioxidant therapies were applied for at least 12 weeks^15–18^. Growth hormone (GH), a peptide secreted by adenohypophysis cells, can reduce OS in some types of cells^19,20^; for this reason, GH has been widely applied to treat pathologies associated with OS, such as burns over large areas, obesity, Alzheimer’s disease and multiple sclerosis^21^. GH bound to the GH receptor (GHR) augments the effects of gonadotropin on GCs and thecal cells, and may improve follicle development and steroidogenesis^22^. Therefore, exogenous GH administration might improve oocyte quality and IVF outcomes among older women and/or patients with poor ovarian response^23^. Weall et al.^24^ found that exogenous GH administration improved oocyte and embryo quality and mitochondrial function in oocytes in such patients. However, Homburg et al.^25^ reported that adjuvant GH therapy did not significantly alter the rates of clinical pregnancy and miscarriage in patients with PCOS. The ability of GH to improve OS and oocyte quality in patients with PCOS has not been assessed in detail. Therefore, this prospective randomized controlled study investigated if GH can significantly improve OS, oocyte quality and IVF outcomes in women with PCOS.

## Results

### Clinical, endocrine, and metabolic characteristics of the study population

**Figure 1:**
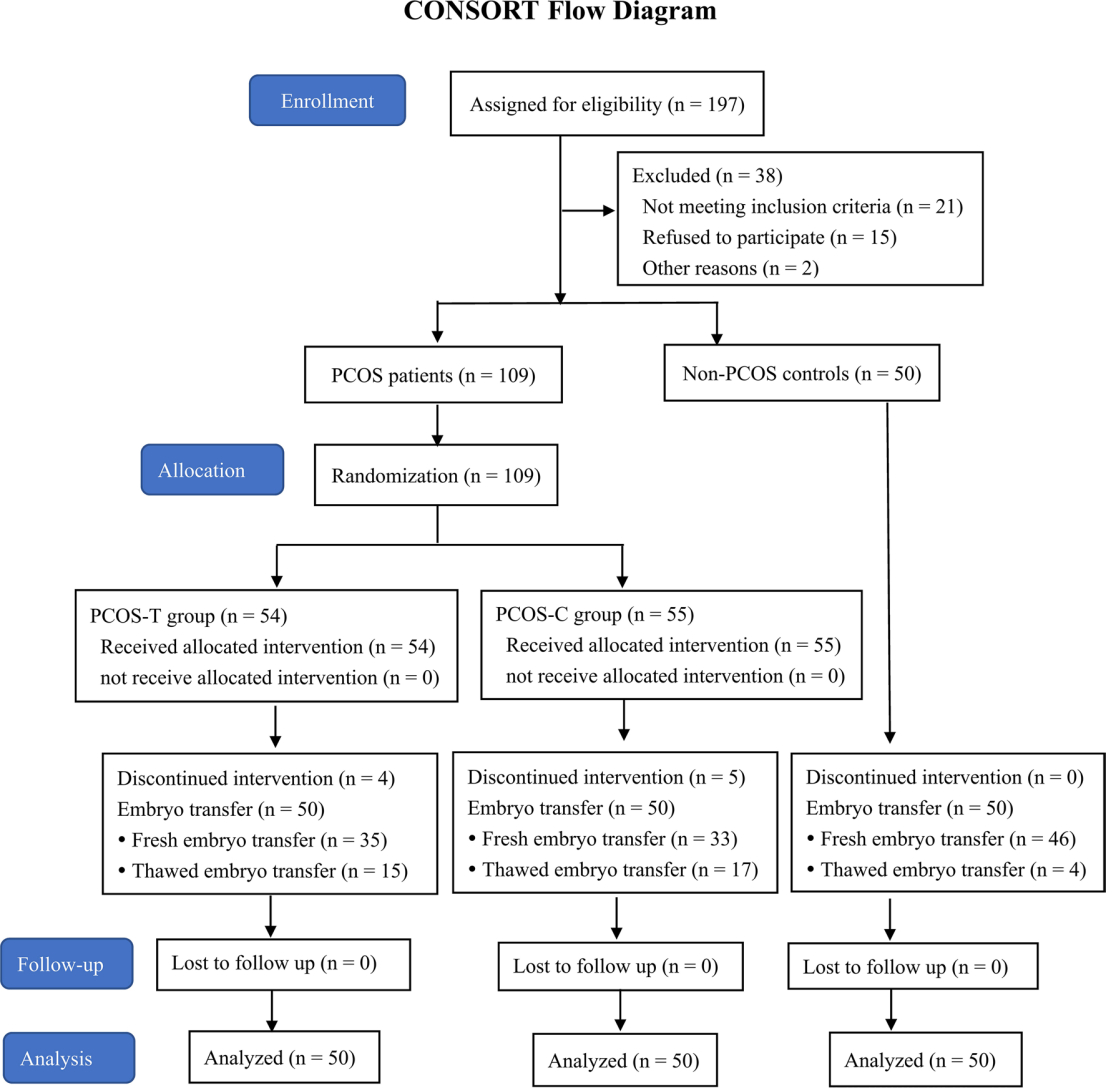
Flow diagram of this randomized controlled trial. Progression from recruitment to completion.

Figure 1 shows the flow of the participants. Among the patients with PCOS, 93, 19, and 49 of them presented with abnormal menstrual cycles, hirsutism, and acne, respectively; and the clinical, endocrine, and metabolic characteristics did not significantly differ between the PCOS-T and PCOS-C groups (*P* > 0.05). The values for irregular menstrual cycles, hirsutism scores, acne scores, waist-to-hip ratio (WHR), antral follicle count (AFC), total testosterone (TT), free androgen index (FAI), luteinising hormone/follicle-stimulating hormone (LH/FSH) ratio, fasting insulin (FINS), and homeostatic model assessment of insulin resistance (HOMA-IR) were significantly higher, whereas sex hormone binding globulin (SHBG) was significantly lower in patients with, compared to without PCOS (controls) (*P* < 0.05 for all; Table 1).

**Table 1:** Clinical, endocrine and metabolic characteristics of study population.

	PCOS-T (n = 54)	PCOS-C (n = 55)	Non-PCOS (n = 50)
Age (years)	28.50 ± 3.51	29.02 ± 3.67	29.66 ± 3.03
Duration of infertility (years)	3.35 ± 2.15	3.98 ± 2.79	3.18 ± 1.95
Irregular menstrual cycle (n)^a,b^	47	46	0
BMI (kg/m^2^)	22.67 ± 2.66	22.44 ± 2.85	21.94 ± 2.55
WHR^a,b^	0.86 ± 0.06	0.86 ± 0.06	0.81 ± 0.05
Hirsutism (n)^a,b^	10	9	0
Acne (n)^a,b^	26	23	0
AFC^a,b^	24.31 ± 5.43	23.47 ± 6.48	15.16 ± 3.81
LH/FSH ratio^a,b^	1.56 ± 0. 86	1.48 ± 0.79	1.01 ± 0.53
E_2_ (pg/mL)	42.75 ± 14.50	47.65 ± 15.01	45.18 ± 14.03
P (ng/mL)	0.46 ± 0.21	0.54 ± 0.21	0.52 ± 0.25
TT (ng/mL)^a,b^	0.67 ± 0.24	0.64 ± 0.29	0.52 ± 0.15
SHBG (nmol/L)^a,b^	46.41 ± 23.12	45.12 ± 27.47	82.50 ± 24.75
FAI^a,b^	11.37 ± 7.21	11.01 ± 4.37	5.17 ± 2.25
FPG (mmol/L)	5.01 ± 0.54	5.02 ± 0.46	4.89 ± 0.47
FINS (micro IU/L)^a,b^	10.36 ± 4.72	10.72 ± 4.35	9.92 ± 5.41
HOMA-IR^a,b^	2.31 ± 1.09	2.41 ± 1.00	1.59 ± 0.76

Data are presented as mean ± SD or number (percentage).

*BMI* body mass index, *WHR* waist-to-hip ratio, *AFC* antral follicle count, *FSH* follicle-stimulating hormone, *LH* luteinizing hormone, *E*_*2*_ oestradiol, *P* progesterone, *TT* total testosterone, *SHBG* sex hormone binding globulin, *FAI* free androgen index, *FPG* fasting plasma glucose, *FINS* fasting insulin, *HOMA-IR* homeostatic model assessment of insulin resistance.

^a^*P* < 0.05 PCOS-T group versus non-PCOS group.

^b^*P* < 0.05 PCOS-C group versus non-PCOS group.

### Controlled ovarian stimulation (COS) and IVF outcomes

The total doses of recombinant FSH (rFSH) were lower in both groups with PCOS, but the oestradiol (E_2_) levels on the trigger day were higher, and more oocytes were retrieved from both groups with PCOS than from the group without PCOS (*P* < 0.05 for all). The numbers of fertilised oocytes (2PN) (8.80 ± 4.84 vs. 6.46 ± 4.61) and cleaved embryos (6.30 ± 4.51 vs. 4.32 ± 3.08) were significantly higher in the PCOS-T group than in the PCOS-C group (*P* < 0.05). The ratio of E_2_ level to the number of follicles with an average diameter ≥ 14 mm on the trigger day and the ratio of E_2_ level to the number of M-II oocytes were not significantly different among the three groups (*P* > 0.05). There were 15, 17 and 4 patients with cancelled fresh embryo transfer in the PCOS-T, PCOS-C and non-PCOS groups, respectively. They all underwent frozen-thawed embryo transfer three months later, resulting in clinical pregnancy for 8, 7 and 2 patients in each group. The numbers of M-II oocytes, higher-quality embryos, implantation rates, and clinical pregnancy rate values tended to be lower, whereas the miscarriage rates tended to be higher in the PCOS-C group than in the PCOS-T group and the group without PCOS, but the differences were not significant (*P* > 0.05). No side effects of GH developed in the PCOS-T group (Table 2).

**Table 2:** COS, IVF outcomes and OS markers in FF.

	PCOS-T (n = 50)	PCOS-C (n = 50)	Non-PCOS (n = 50)
**Clinical outcomes**			
Total dose of rFSH (IU)^a,b^	1791.74 ± 400.29	1821.02 ± 476.91	2119.75 ± 648.21
Duration of COS (d)	10.48 ± 1.87	10.56 ± 1.79	10.26 ± 1.69
E_2_ on trigger day (pg/mL)^a,b^	3903.74 ± 2532.36	4011.30 ± 2887.39	2632.99 ± 1217.87
E_2_/No. follicles ≥ 14 mm on trigger day (pg/mL)	268.27 ± 46.57	279.65 ± 52.33	259.87 ± 45.38
E_2_/No. MII (pg/mL)	321.56 ± 51.18	335.47 ± 61.62	317.69 ± 59.36
No. severe OHSS	0	0	0
**Embryological and pregnancy outcomes**			
No. oocytes retrieved^a,b^	15.52 ± 7.96	14.64 ± 6.49	11.72 ± 4.99
No. MII	12.30 ± 6.80	10.02 ± 6.48	9.94 ± 5.30
No. fertilized oocytes (2PN)^c^	8.80 ± 4.84	6.46 ± 4.61	7.34 ± 3.91
No. cleaved embryos^c^	6.30 ± 4.51	4.32 ± 3.08	5.02 ± 3.62
No. higher-quality embryos	3.32 ± 3.05	2.46 ± 2.25	3.00 ± 2.96
No. embryos transferred	1.74 ± 0.44	1.78 ± 0.42	1.78 ± 0.42
Implantation rate (%)	36.78% (32/87)	29.21% (26/89)	33.71% (30/89)
Clinical pregnancy rate/ET (%)	54.00% (27/50)	42.00% (21/50)	50.00% (25/50)
Early miscarriage rate (%)	7.41% (2/27)	9.52% (2/21)	4.00% (1/25)
Multiple pregnancy rate (%)	18.50% (5/27)	23.80% (5/21)	20.00% (5/25)
**FF oxidative status**			
FF MDA (µmol/L)	2.50 ± 1.25	2.51 ± 0.82	2.19 ± 0.70
FF SOD (U/mg prot)	13.81 ± 1.50	13.81 ± 1.53	13.90 ± 2.11
FF TAC (mmol Trolox Eq/L)	0.68 ± 0.09	0.68 ± 0.08	0.64 ± 0.10
FF TOS (µmol H_2_O_2_ Eq/L)^b,c^	8.16 ± 1.88	10.48 ± 2.68	8.38 ± 2.35
FF OSI^b,c^	12.34 ± 3.90	15.59 ± 5.32	13.10 ± 4.78

Data are presented as mean ± SD or number (percentage).

*2PN* number of two pronuclear zygotes, *OHSS* ovarian hyperstimulation syndrome, *MDA* malondialdehyde, *SOD* superoxide dismutase, *TAC* total antioxidant capacity, *TOS* total oxidant status, *OSI* oxidative stress index.

^a^*P* < 0.05 PCOS-T versus non-PCOS.

^b^*P* < 0.05 PCOS-C versus non-PCOS.

^c^*P* < 0.05 PCOS-T versus PCOS-C.

### OS markers

Serum malondialdehyde (MDA), superoxide dismutase (SOD), and total antioxidant capacity (TAC) did not significantly differ among the three groups before COS (*P* > 0.05). The total oxidant status (TOS) and the OS index (OSI) were increased in patients with PCOS compared with those without PCOS (*P* < 0.05) but did not significantly differ between the PCOS-T and PCOS-C groups (*P* > 0.05). The OS markers on the trigger day also did not significantly differ among the three groups (*P* > 0.05). However, compared with the baseline values in all groups, the TOS and OSI increased, and the TAC decreased (*P* < 0.05; Table 3).

**Table 3:** Serum OS markers at baseline and on the trigger day.

	PCOS-T (n = 50)			PCOS-C (n = 50)			Non-PCOS (n = 50)		
	Baseline	Trigger day	Change	Baseline	Trigger day	Change	Baseline	Trigger day	Change
MDA (µmol/L)	4.87 ± 0.95	5.08 ± 1.24	0.20 ± 0.48	4.71 ± 1.02	5.13 ± 1.02	0.42 ± 0.24	4.67 ± 1.02	4.70 ± 0.78	0.33 ± 0.29
SOD (U/mg prot)	14.22 ± 1.39	15.23 ± 2.56	1.02 ± 1.20	14.49 ± 1.92	14.96 ± 1.89	0.47 ± 0.56	14.23 ± 2.04	15.11 ± 2.23	0.89 ± 0.63
TAC (mmol Trolox Eq/L)^a^	0.70 ± 0.15	0.62 ± 0.15	-0.08 ± 0.06	0.72 ± 0.10	0.60 ± 0.17	-0.11 ± 0.08	0.70 ± 0.11	0.59 ± 0.19	-0.11 ± 0.08
TOS (µmol H_2_O_2_ Eq/L)^a^	19.71 ± 7.96	24.05 ± 9.82	4.34 ± 2.99	19.40 ± 7.27	24.24 ± 10.09	4.85 ± 3.69	15.15 ± 5.89	22.83 ± 7.98	7.68 ± 3.21
OSI^a^	29.02 ± 11.76	39.18 ± 13.58	10.15 ± 7.75	27.29 ± 9.81	42.97 ± 17.09	15.68 ± 11.91	21.60 ± 7.57	40.78 ± 12.80	16.18 ± 9.23

Data are presented as mean ± SD.

*MDA* malondialdehyde, *SOD* superoxide dismutase, *TAC* total antioxidant capacity, *TOS* total oxidant status, *OSI* oxidative stress index.

^a^*P* < 0.05 parameter on the trigger day versus baseline.

The values for MDA, SOD, and TAC in FF did not significantly differ among the three groups on the trigger day (*P* > 0.05), whereas the TOS and OSI were significantly higher in the PCOS-C group than in the PCOS-T group and in the group without PCOS (*P* < 0.05; Table 2).

### Correlations between basal serum OS markers with metabolic and androgen parameters

In patients with PCOS, the TOS and OSI were positively correlated with the HOMA-IR, FINS and TT levels and negatively correlated with the SHBG levels (*P* < 0.05). No significant correlations were observed between the MDA, SOD and TAC levels with metabolic and androgen parameters (*P* > 0.05; Table 4).

**Table 4:** Correlations between basal serum OS markers with metabolic and androgen parameters.

Serum OS markers	FINS (micro IU/L)		HOMA-IR		TT (ng/mL)		SHBG (nmol/L)	
	*r*	*P*	*r*	*P*	*r*	*P*	*r*	*P*
MDA (µmol/L)	0.04	0.69	0.02	0.81	0.15	0.13	− 0.08	0.43
SOD (U/mg prot)	− 0.08	0.41	− 0.08	0.41	− 0.04	0.67	0.14	0.15
TAC (mmol Trolox Eq/L)	− 0.06	0.53	− 0.06	0.53	− 0.13	0.18	0.08	0.40
TOS (µmol H_2_O_2_ Eq/L)	0.23	0.02	0.23	0.02	0.22	0.02	− 0.21	0.03
OSI	0.33	0.00	0.32	0.00	0.28	0.00	− 0.28	0.00

*MDA* malondialdehyde, *SOD* superoxide dismutase, *TAC* total antioxidant capacity, *TOS* total oxidant status, *OSI* oxidative stress index, *FINS* fasting insulin, *HOMA-IR* homeostatic model assessment of insulin resistance, *TT* total testosterone, *SHBG* sex hormone binding globulin, *r* Pearson correlation coefficient.

### Mitochondrial function of GC

**Figure 2:**
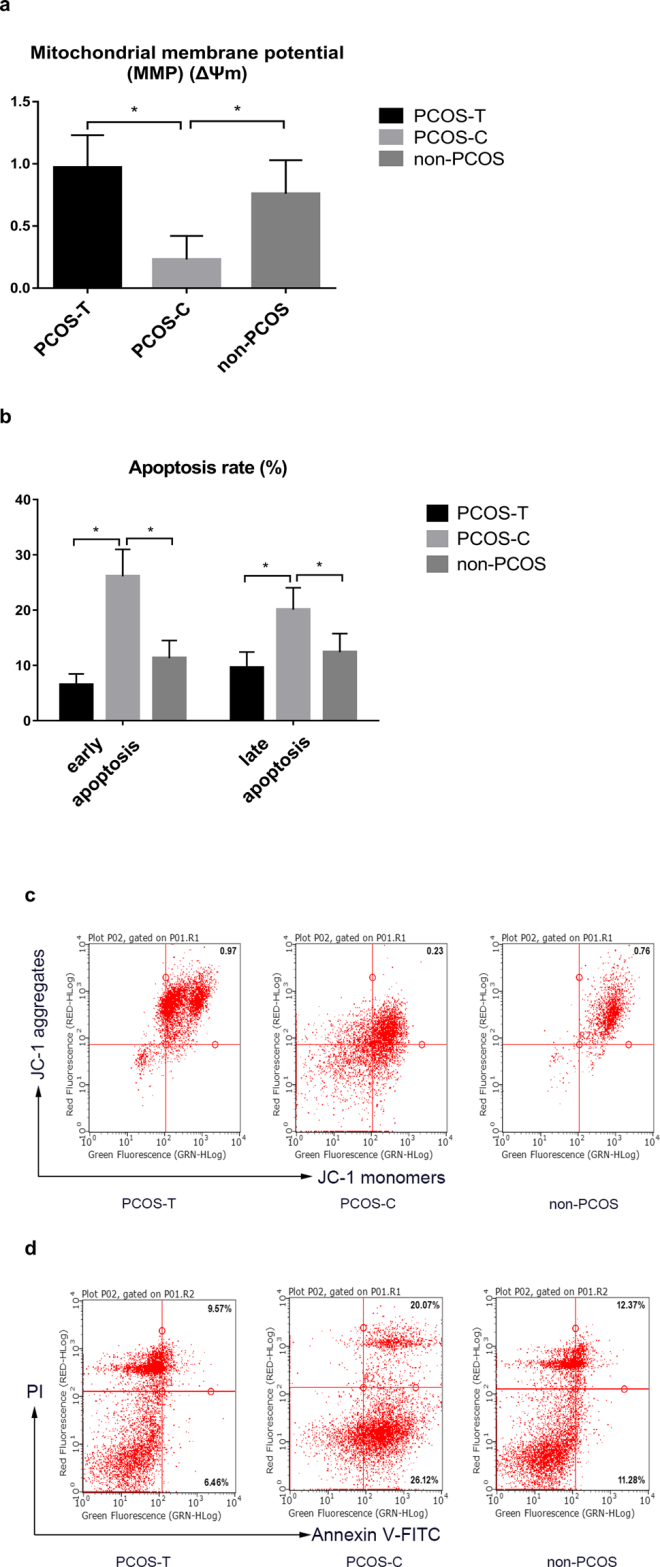
Flow cytometric dot plots of effects of GH on mitochondrial function in granulosa cells. (**a**, **c**) The MMP in GCs was lower in the PCOS-C group than in the non-PCOS control group, as shown by reduced JC-1 aggregates red fluorescence. The MMP was greater in the PCOS-T group than in the PCOS-C group (*P* < 0.05). (**b**, **d**) The numbers of early and late apoptotic cells were increased in the PCOS-C group compared with those in the non-PCOS and PCOS-T groups (*P* < 0.05). *GH* growth hormone, *MMP* mitochondrial membrane potential, *PCOS* polycystic ovary syndrome, *PI* propidium iodide, *FITC* fluorescein isothiocyanate. *P < 0.05 denotes statistical significance between groups.

The mitochondrial membrane potential (MMP) was significantly higher (0.97 ± 0.26 vs. 0.23 ± 0.19), whereas the early and late apoptosis rates were significantly lower (6.46% vs. 26.12% and 9.57% vs. 20.07%, respectively) in the PCOS-T group than in the PCOS-C group (*P* < 0.05 for all). The MMP was significantly lower (0.23 ± 0.19 vs. 0.76 ± 0.27) and the early and late apoptosis rates were significantly higher (26.12% vs. 11.28%, and 20.07% vs. 12.37%) in the PCOS-C group than in the group without PCOS (*P* < 0.05 for all; Fig. 2). Mitochondrial function was a main indicator in this study. A power calculation based on sample size and mitochondrial results was performed by Power Analysis and Sample Size (PASS) 11. The result showed that a statistical power of 0.8692 was achieved in the study for the early apoptosis rate between PCOS-T group and PCOS-C group (difference = 0.1965, control group proportion = 0.0646, significance level = 0.05).

## Discussion

This prospective, randomized, controlled study found increased OS and dysfunctional mitochondria in the GCs of patients with PCOS undergoing IVF. However, we also determined that GH could lower the TOS and OSI in FF and improve the mitochondrial function of GCs. We also found more fertilised oocytes and cleaved embryos in the PCOS-T group than in the PCOS-C group, whereas the rates of implantation, miscarriage, and clinical pregnancy did not significantly differ among all groups.

Although numerous studies have identified OS in patients with PCOS, the experimental findings of specific OS markers are contradictory^9,26–28^. We found significantly higher TOS and OSI levels in serum and FF in patients with PCOS than in those without PCOS, whereas the MDA, TAC, and SOD levels did not significantly differ. However, Wang et al.^26^ reported higher values for MDA along with lower values for SOD and TAC. Others have found that the SOD and TAC levels increase to compensate for OS^27,28^. These inconsistent results might be caused by relatively small samples and differing methodologies. Analysis from our findings, MDA is a product of lipid peroxidation, but TOS represents different oxidant molecules’ additive effects^29^, ROS/RNS molecules other than MDA might have substantially contributed to the higher TOS. However, because of limited funding and specimens, we did not measure the levels of other ROS/RNS markers. Furthermore, TAC represents the ability to eliminate free radicals^30^, and OSI (the ratio of TOS/TAC) can evaluate the balance of oxidation and anti-oxidation^31^. We found a slightly higher TAC in patients with PCOS than in those without PCOS, but the difference was not significant. Thus, the absolute TOS was higher, but the ability to eliminate free radicals was relatively insufficient in patients with PCOS. This finding agreed with the notion that OS is extant in PCOS because physiological antioxidants are insufficient to outweigh the excess ROS^2^. In addition, we found that the OS markers in FF and serum concurred. FF originates from blood diffusion and from secretion from GCs and oocytes; thus, OS markers in FF reflect the systemic OS status and that of the ovaries^32^. Through correlation analysis, we found that the basal serum TOS and OSI were both positively correlated with HOMA-IR, FINS and TT levels and negatively correlated with SHBG. These results were consistent with those of other studies showing that the pathophysiology of PCOS, such as hyperandrogenaemia and IR, was associated with OS^11–13^. The above evidence indicated the OS status of patients with PCOS, which suggests that antioxidant therapy might be beneficial.

We found that GH reduced the FF TOS by 2.32 mmol H_2_O_2_ Eq/L and the OSI by 3.25 in patients with PCOS. However, we did not find the same effects on the systemic OS status because serum OS markers on the trigger day did not significantly differ. Some possible explanations for this finding are as follows. Ten days of GH administration might not be sufficient to alleviate systemic OS. Furthermore, systemic OS is closely associated with obesity, IR, and hyperandrogenism^11^. Patients with PCOS in this study exhibited abnormal endocrine and metabolic statuses; therefore, GH alone might not be able to alleviate systemic OS. The antioxidant effects of GH seem to be exerted mainly in follicles^24^.

Meanwhile, compared with baseline values in all groups, the serum TOS and OSI were significantly increased, whereas the TAC was significantly decreased on the trigger day. The accumulation of ROS during COS disrupts the oxidant-antioxidant balance and leads to OS^7^. Other studies have also reported the OS state and GC apoptosis during COS, which might reduce the oocyte development potential^7,33^. Such findings provide another rationale for treatment with antioxidants in IVF.

With respect to the ovarian OS status, we found that the early and late apoptosis rates of GCs approximately doubled, and the MMP decreased by 70% in patients with PCOS. GCs, which provide nutrients and factors that enable oocyte maturation via intimate cross-talk, are steroidogenic^7^. Mitochondria in GCs are critical for oocyte development and competence and for glucose metabolism^34^. OS leads to GC apoptosis in patients with PCOS by reducing mitochondrial DNA (mtDNA) copy numbers and impairing mitochondrial oxidative metabolism^7^. Moreover, COS induces ROS production in GCs, which aggravates mitochondrial dysfunction and GC apoptosis^28^.

GH might improve mitochondrial function in some types of cells, including oocytes, vascular endothelium, and skeletal muscle cells^24,35^. We found here that GH apparently improved the MMP over three-fold and decreased GC apoptosis by > 50%. The mechanism of how GH improves mitochondrial function is not fully understood. Activating the components of mitochondrial fuel delivery and enhancing ATP generation might be one mechanism^35^.

FF and GCs constitute an important microenvironment for oocyte development. Along with alleviating OS, GH administration resulted in significantly more fertilised oocytes and cleaved embryos. The number of higher-quality embryos was improved by 0.86 but this improvement was not significant. Others have found that GH significantly elevates the number and quality of oocytes^36^. However, conclusions from these results should be drawn with caution because of the small sample size.

The poorer IVF outcomes in patients with PCOS than in those without PCOS is mainly due to poorer oocyte quality in patients with PCOS^2^. Along with more oocytes with better quality, we found that GH increased the implantation rate and clinical pregnancy rate and lowered the miscarriage rate, but the difference was not significant. However, Homburg et al.^25^ reported that adjuvant GH therapy did not significantly alter the rates of clinical pregnancy and miscarriage in patients with PCOS. However, caution is advised when analysing these results due to the relatively small sample size.

This study has some limitations. We measured only several OS markers due to limited funding and specimens. Moreover, the sample size was too small to analyse outcomes according to the PCOS subtype.

In summary, our study revealed that GH combined with gonadotropin significantly improved mitochondrial dysfunction in GCs and oocyte quality in PCOS women. Because the precise mechanism through which GH influences OS status remains obscure, further basic investigations at the cellular level in vitro are needed to define the involved mechanisms.

## Materials and methods

### Ethics statements

This prospective, randomized, open-label study was registered in the Chinese Clinical Trial Registry Centre (Registration No. ChiCTR1800019437) before the first patient was enrolled, and was approved by the Chinese Ethics Committee of Registering Clinical Trials. Written informed consent to participate in the study was obtained from all participants. All procedures in this study complied with the ethical standards of the relevant national and institutional committees on human experimentation and with the Helsinki Declaration of 1975 (2013 revision).

The primary outcomes included markers of OS in serum and FF, and the secondary outcomes were mitochondrial function in GCs and IVF-ET outcomes. The sample size was calculated based on differences in serum TOS^37^ between patients with and without PCOS. We used 6.80 µmol H_2_O_2_ Eq/L as the mean difference (d) and 7.6 µmol H_2_O_2_ Eq/L as the SD for TOS as the key variable^37^. Each group contained 43 participants with an α of 0.05 and a β error of 0.1 (power = 90%). Assuming a dropout of seven participants per group with a dropout rate of 14%, the final sample size was 50 participants per group.

### Study population

Between November 2018 and November 2019 at the Department of Reproductive Medicine, West China Second University Hospital, Sichuan University, 109 patients (aged 20–40 years) diagnosed with PCOS according to the Rotterdam criteria^38^ and undergoing IVF treatment were enrolled in this study. The patients were randomly assigned (using computer-generated random numbers) to undergo treatment with (PCOS-T, n = 54) or without (PCOS-C, n = 55) GH. Patients were excluded from the study if they met any of the following criteria: (1) hydrosalpinx; (2) congenital uterine malformations and/or endometrial disease, tuberculosis, hyperplasia; (3) systemic lupus erythematosus and/or sicca syndrome; (4) uncontrolled endocrinopathy such as diabetes, hyperthyroidism, hypothyroidism, and hyperprolactinemia; (5) cigarette smoking and/or alcohol consumption; (6) supplementation with vitamin E, vitamin C, or CoQ10, which influence OS markers. Infertile women (aged 20–40 years) who underwent IVF-ET due to tubal issues were recruited as non-PCOS controls (n = 50), and the inclusion criterion for control patients required the absence of all the Rotterdam criteria. The exclusion criteria for this group were the same as those for the patients with PCOS. Each participant had read and signed informed consent before being enrolled in the study. In the PCOS-T group, three patients discontinued recombinant human GH (rhGH) during COS because of the cost, and one patient declined further participation. In the PCOS-C group, two patients abandoned treatment because they left Chengdu City, and three patients declined further participation. No patients in the non-PCOS group dropped out. Therefore, 50 participants per group completed the study.

Medical histories regarding menstrual cycle regularity, duration of infertility, and treatment were collected from all participants. Physical examinations included measurements of height, body weight (without shoes and heavy clothing), waist circumference at the midpoint between the lower rib margin and the top of the iliac crest at end exhalation, and hip circumference at the level of the greater trochanter. Body mass index (BMI) was calculated as weight divided by height squared (kg/m^2^). The WHR was calculated as waist circumference divided by hip circumference. Blood samples were collected from elbow veins after an overnight fast on the same day that the participants were assessed by transvaginal ultrasound on days 2–3 of menstruation or progestin-induced withdrawal bleeding. The blood samples were separated by centrifugation at 2132 × *g* for 15 min at 4 °C within 2 h, and serum was stored at – 80 °C.

### Regimen for COS and IVF

The COS protocol for all patients was the gonadotropin-releasing hormone (GnRH) antagonist protocol. rFSH (Gonal-F; Merck-Serono KGaA., Darmstadt, Germany) was started from day 2 of the menstrual cycle; then gonadotropin (Gn) doses were adjusted according to follicular growth. When serum E_2_ concentration reached 300 pg/mL or when the lead follicle grew to a diameter of 13–14 mm, cetrorelix (Cetrotide; Merck-Serono KGaA.) was administered. On the same day as the rFSH administration, only patients in the PCOS-T group were subcutaneously injected with 4 IU/day of rhGH for pharmaceutical use (Jintropin, Changchun GeneScience Pharmaceutical Co., Ltd., Changchun, Jilin, China) until the trigger day. Recombinant human chorionic gonadotropin (hCG) (Ovitrelle; Merck-Serono KGaA.) was administered as the trigger when the diameters of at least two follicles reached ≥ 18 mm, and blood samples were collected on the same day prior to hCG administration. After 36 h, each follicle was measured immediately prior to its aspiration; then, oocytes were retrieved under transvaginal ultrasound guidance, and follicle flushing was not performed.

FF and GCs from each patient were separately collected and considered as one sample. FF derived from follicles with a diameter ≥ 16 mm in one case were pooled together and immediately centrifuged at 700 × *g* for 5 min at room temperature, and the supernatant was stored at – 80 °C. The precipitates were suspended with 2 mL of remaining FF and then gently layered onto 3 mL of 50% lymphocyte separation medium (Beijing Solarbio Science and Technology Corporation, Beijing, China) and centrifuged at 700 × *g* for 10 min at room temperature to remove red blood cells and debris. GCs were collected from the layer at the interface of the gradient and washed twice with 5 mL of PBS (Nanjing KeyGen Biotech. Co., Ltd**.,** Nanjing, Jiangsu, China), and mitochondrial function was immediately examined.

After fertilization in vitro, cultured embryos were evaluated on day 3, and according to the number of blastomeres and the degree of fragmentation, the higher-quality embryos were categorised as grades A/B^39^. One or two higher-quality day 3 embryos were transferred. Luteal phase support with intramuscular (i.m.) injections of progesterone (P) at 60 mg/day started on retrieval day. Serum levels of hCG, E_2_, and P were measured at 14 days after ET and hCG values > 5 IU/mL were considered positive. Clinical pregnancy was defined as a gestational sac containing an embryo with normal cardiac activity. Early miscarriage was defined as loss of pregnancy before gestational week 12. Rates of implantation, clinical pregnancy, and miscarriage were calculated. Ovarian hyperstimulation syndrome (OHSS) was diagnosed according to Navot D et al.^40^. Mild OHSS was defined as patients with at least one symptom consistent with OHSS (nausea, vomiting or diarrhoea) and a maximal ovarian diameter of 5–12 cm. Moderate OHSS was designated as patients with ultrasonic evidence of ascites (sub-hepatic ascites or post-cervical ascites ≥ 10 mm). Severe OHSS was defined as patients with clinical evidence of ascites or hydrothorax or breathing difficulties, or any of the following conditions: haematocrit > 45%; creatinine > 90 µmol/L; alanine aminotransferase > 45 U/L; or oliguria, renal failure, thromboembolic phenomena or adult respiratory distress syndrome^40^. The fresh embryo transfer was cancelled when the patient met any of the following criteria: the number of oocytes retrieved was > 18; the patient was diagnosed with moderate or severe OHSS; a P level was > 1.5 ng/mL on the trigger day; or there was abnormal endometrial thickness (< 7 mm or > 15 mm on the trigger day). Only the first embryo transfer cycle (fresh or frozen-thawed embryo transfer) was analysed in this study. The patients underwent frozen-thawed embryo transfer at least three months later. The physicians making treatment decisions for women with PCOS, such as gonadotropin dosing, trigger timing and oocyte retrieval, embryo evaluation and embryo transfer, were blinded as to study arm (rhGH or not) (Supplementary Information).

### Experimental procedures

Plasma glucose was measured using the hexokinase method (Roche Diagnostics GmbH, Mannheim, Germany). E_2_, P, TT, LH, FSH, thyroid-stimulating hormone (TSH), SHBG, and insulin levels were measured using chemiluminescent immunoassays (Siemens Healthcare Diagnostic Inc., Massachusetts, USA). The sensitivity of the TT assay kit was 0.07 ng/mL. The FAI was calculated as TT (nmol/L)/SHBG (nmol/L) × 100. The HOMA-IR index was calculated as fasting glucose (mmol/L) × FINS (micro IU/L)/22.5^41^. The intra- and inter-assay coefficients of variation for these values were < 5% and < 10%, respectively.

Serum MDA concentrations (µmol/L) were determined using micro-MDA detection kits (NanJing Jiancheng Bioengineering Institute Co. Ltd., Nanjing, Jiangsu, China) and ultraviolet spectrophotometry (Shanghai Meipuda Instrument Co., Ltd., Shanghai, China) was performed at 532 nm. SOD was determined using SOD kits (NanJing Jiancheng Bioengineering Institute Co. Ltd.) and spectrophotometry at 450 nm^26^. Using Trolox [(±) 6-hydroxy-2,5,7,8-tetramethylchroman-2-carboxylic acid] as a standard, the TAC (mmol Trolox Equiv./L) was determined based on the decolourisation of 2,2′-azinobis (3-ethylbenzothiazoline-6-sulphonate) (ABTS). Changes in the colour were measured as absorbance at 420 nm^30^. Using hydrogen peroxide (H_2_O_2_) as a standard, the TOS (µmol H_2_O_2_ Eq/L) was measured as the oxidation of ferrous to ferric ions by oxidant species in acidic medium. Ferric ions complexed with xylenol orange were measured at 594 nm^31^. These semi-automatic microplate colorimetric methods are simple, stable, reliable, and sensitive for determination of TAC and TOS. The OSI was calculated as the ratio of the TOS to that of the TAC. All OS parameters were detected in duplicate. Serum samples from healthy volunteers were pooled for quality control. All OS markers in FF and serum were similarly determined. The intra- and inter-assay coefficients of variations of these parameters were all < 5% and < 10%, respectively.

Apoptosis of GCs was detected using Annexin V-FITC Apoptosis Detection Kits (KeyGEN Bio TECH Co., Ltd.) using flow cytometry (MilliporeSigma Co., Ltd., Burlington, MA, USA). The MMP of GCs was examined using JC-1 Apoptosis Detection Kits (KeyGEN Bio TECH Co., Ltd.). GCs were cultured with JC-1 staining solution for 20 min at 37 °C without exposure to light and then washed twice with incubation buffer. JC-1 accumulates in functional mitochondria with high ΔΨm and forms aggregates that emit red fluorescence. When mitochondrial transmembrane potential is depolarized with low ΔΨm, JC-1 releases from the mitochondria and forms monomers that emit green fluorescence. The fluorescence intensity of JC-1 was detected using flow cytometry. The ratios of red/green fluorescence were calculated to characterise the MMP^42^.

### Statistical analysis

All data were statistically analysed using SPSS 17.0 software (SPSS Inc., Chicago IL, USA). Continuous variables are expressed as means ± standard deviations (SDs). The normality of the data distribution was assessed using Kolmogorov–Smirnov tests. Within-group pre- and post-treatment parameters were compared using Student–Newman–Keuls tests for continuous variables with a normal distribution. Between-group comparisons were assessed using one-way ANOVA with post hoc Bonferroni tests. Pearson’s correlation was applied to analyse the relationship between basal serum OS markers with metabolic and androgen parameters. Categorical data were compared using Chi-squared tests. Two-tailed* P* values < 0.05 were considered statistically significant.

### Ethics approval

Approval was obtained from the Chinese Ethics Committee of Registering Clinical Trials (ChiECRCT-20180176). The procedures used in this study adhere to the tenets of the Declaration of Helsinki.

### Consent to participate

Written informed consent was obtained from each participant.

## Supplementary information

Supplementary Information 1.

Supplementary Information 2.

## Data Availability

The datasets used and/or analyzed during the current study are available from the corresponding author on reasonable request.
