# Supplementary material for: Growth hormone alleviates oxidative stress and improves oocyte quality in Chinese women with polycystic ovary syndrome: a randomized controlled trial
Source: Sci Rep. 2020 Oct 30;10:18769. doi: 10.1038/s41598-020-75107-4 (PMC7599233; doi:10.1038/s41598-020-75107-4)
Supplement: Supplementary file 2 — Supplementary Information 2. [file 41598_2020_75107_MOESM2_ESM.docx]

**Research protocol: part 1**

**Project summary****:** Oxidative stress (OS) is associated with poor oocyte quality and poor *in vitro* fertilization and embryo transfer (IVF-ET) outcomes for patients with polycystic ovary syndrome (PCOS). Growth hormone (GH) can function reduce OS in some types of cells. Therefore, this prospective, randomized study investigated whether GH can significantly improve OS and oocyte quality in women with PCOS. This study enrolled 100 and 50 patients with and without PCOS (controls), respectively, between November 2018–November 2019. The patients with PCOS were randomly assigned to receive treatment with GH (PCOS-T) or not (PCOS-C). The primary outcome included markers of oxidative stress (OS) in serum and follicle fluid (FF), and secondary outcomes were mitochondrial function in granulosa cells (GC) and IVF-ET outcomes. The PCOS groups showed higher basal serum total oxidant status (TOS) and the oxidative stress index (OSI) levels. The follicle fluid (FF) TOS and OSI, and granulosa cells (GC) apoptosis rate were significantly higher, whereas GC mitochondrial membrane potential was significantly lower in PCOS-C group compared with PCOS-T and non-PCOS control groups (P < 0.05). Significantly more oocytes were fertilised and embryos were cleaved in PCOS-T group than in PCOS-C group (P < 0.05). GH also improved the rates of implantation and clinical pregnancy; however, the difference did not reach significance (P > 0.05). This study showed that GH alleviated OS and improved GC mitochondrial dysfunction and oocyte quality in patients with PCOS.

**General information**

**Title:** Growth hormone alleviates oxidative stress and improves oocyte quality in Chinese women with polycystic ovary syndrome: A randomized controlled trial

**Protocol number and date:** ChiCTR1800019437, between November 2018 and November 2019.

**Funding:** The Key Research and Development project of Science and Technology Bureau of Sichuan (2019YF S0406, Sichuan, China) and the Technology Innovation Project of Science and Technology Bureau of Chengdu (2018-YF05-00247-SN, Chengdu, Sichuan, China).

**Investigators:** Yan Gong, Shan Luo, Ping Fan, Song Jin, Huili Zhu, Tang Deng, Yi Quan, and Wei Huang.

**Investigators' responsibilities:** Yan Gong designed the study and wrote the manuscript. Shan Luo participated in sample collection and data analysis. Ping Fan contributed to laboratory instruction and revision of the article. Song Jin, Huili Zhu, Tang Deng and Yi Quan contributed to sample collection. Wei Huang contributed to design and revise the article.

**Address and telephone numbers of the research sites:**

1 Department of Obstetrics and Gynecology, West China Second University Hospital of Sichuan University, Chengdu, Sichuan, P.R. China. Tel: +86-28-88570156.

2 Key Laboratory of Birth Defects and Related Diseases of Women and Children, Ministry of Education, Chengdu, Sichuan, P.R. China. Tel: +86-28-85503259.

3 Reproductive Medicine Center, Sichuan Provincial Hospital for Women and Children, Chengdu, Sichuan, People's Republic of China. Tel: +86-28-65978272.

**Background information**

Oxidative stress (OS) is associated with poor oocyte quality and poor in vitro fertilization and embryo transfer (IVF-ET) outcomes for patients with polycystic ovary syndrome (PCOS)^1^. Growth hormone (GH) can function reduce OS in some types of cells^2, 3^. The ability of GH to improve OS and oocyte quality in patients with PCOS has not been assessed in detail.

**Study goals and objectives**

To investigate whether GH can significantly improve OS and oocyte quality in women with PCOS. The primary outcome included markers of OS in serum and follicle fluid (FF), and secondary outcomes were mitochondrial function in granulosa cells (GC) and IVF-ET outcomes.

**Study design**

This prospective, randomized, controlled study enrolled 100 and 50 patients with and without PCOS (controls), respectively, who underwent conventional IVF-ET. Patients with PCOS were diagnosed according to the Rotterdam criteria^4^. Patients were excluded from the study if they met any of the following criteria: (1) hydrosalpinx; (2) congenital uterine malformations and/or endometrial disease, tuberculosis, hyperplasia; (3) systemic lupus erythematosus and/or sicca syndrome; (4) uncontrolled endocrinopathy such as diabetes, hyperthyroidism, hypothyroidism, and hyperprolactinemia; (5) cigarette smoking and/or alcohol consumption; and (6) supplementation with vitamin E, vitamin C, and CoQ10 that influence OS markers. The patients were randomly assigned (using computer-generated random numbers) to undergo treatment with (PCOS-T, n=50) or without (PCOS-C, n=50) GH. Other than regular gonadotropin, the PCOS-T group was subcutaneously injected with 4 IU/day of recombinant human growth hormone (Jinsai Pharmaceutical Co., Ltd., Changchun, Jilin, China) until the trigger day. The commercial reagent of GH was used in this study, therefore without blinding method.

**Methodology**

The controlled ovarian stimulation (COS) protocol for all patients was GnRH antagonist. Rates of implantation, clinical pregnancy, and miscarriage were calculated. After IVF, cultured embryos were evaluated on day 3 according to the number of blastomeres and the degree of fragmentation. The higher quality embryos were categorized as grades A/B^5^. One or two higher-quality D3 embryos were transferred. Ovarian hyperstimulation syndrome (OHSS) was diagnosed according to Navot D et al^6^. All embryos were frozen for frozen-thawed embryo transfer three months later for patients at high risk of OHSS. Markers of OS in serum and FF in this study included malondialdehyde (MDA), superoxide dismutase (SOD), total antioxidant capacity (TAC), total oxidant status (TOS) and oxidative stress index (OSI). MDA and SOD were determined using ultraviolet spectrophotometry^7^. TAC and TOS were determined using the semi-automatic microplate colorimetric methods^8, 9^. OSI was calculated as the ratio of TOS to TAC. The apoptosis and mitochondrial membrane potential of GC were detected using flow cytometry. Figure 1 shows the ﬂow of the participants (CONSORT flow diagram) (between November 2018–November 2019).

**Safety considerations**

No side effects developed in all participants.

**Follow-up**

Serum levels of human chorionic gonadotropin (hCG) was measured at 14 days after ET and hCG values >5 IU/mL were considered positive. Otherwise it was negative and the data collection was completed. Clinical pregnancy was defined as a gestational sac containing an embryo with normal cardiac activity. Early miscarriage was deﬁned as loss of pregnancy before gestational week 12. If the patient was pregnant, the data collection was continued until gestational week 12 or early miscarriage occurred.

**Data management and statistical analysis**

Sample size was calculated based on differences in serum TOS^10^ between patients with and without PCOS. We used 6.80 µmol H_2_O_2_ Eq/L as the mean difference (d) and 7.6 µmol H_2_O_2_ Eq/L as the SD for TOS as the key variable. Each group contained 43 participants with an α of 0.05 and a β error of 0.1 (power = 80%). Assuming a dropout of seven participants per group, the final sample size was 50 participants per group.

All data were statistically analysed using SPSS 17.0 software (SPSS Inc., Chicago IL, USA). Continuous variables are expressed as means ± standard deviation (SD). The normality of the data distribution was assessed using Kolmogorov–Smirnov tests. Within-group pre- and post- treatment parameters were compared using Student-Newman-Keuls tests for continuous variables with normal distribution. Between-group comparisons were assessed using one-way ANOVA with post hoc Bonferroni tests. Categorical data were compared using Chi-squared tests. Two-tailed P values <0.05 were considered statistically significant.

**Quality assurance**

There is a standard data collection and management system in the Department of Reproductive Medicine.

**Expected outcomes of the study**

This study showed that GH combined with gonadotropin significantly alleviated OS status in ovaries, improved mitochondrial dysfunction in GC and oocyte quality in patients with PCOS. The results will be beneficial for the patients with PCOS undergoing IVF.

**Dissemination of results and publication policy**

Wei Huang will take the lead in publication.

**Duration of the project**

November 2018-December 2018, design and preliminary experiment of the study.

January 2019-November 2019, enrollment the patients and experimental study.

December 2019-January 2020, writing the revising the manuscript.

**Problems anticipated**

None.

**Project management**

Yan Gong designed the study and wrote the manuscript. Shan Luo participated in sample collection and data analysis. Ping Fan contributed to laboratory instruction and revision of the article. Song Jin, Huili Zhu, Tang Deng and Yi Quan contributed to sample collection. Wei Huang contributed to design and revise the article.

**Ethics**

Approval was obtained from the Chinese Ethics Committee of Registering Clinical Trials (ChiECRCT-20180176). Written informed consent to participate in the study was obtained from all participants. All procedures in this study complied with the ethical standards of the relevant national and institutional committees on human experimentation and with the Helsinki Declaration 1975 (2013 revision).

**Informed consent forms**

In the supplementary materials.

**References**

1. Zhao, H.; Zhao, Y.; Li, T.; Li, M.; Li, J.; Li, R.; Liu, P.; Yu, Y.; Qiao, J., Metabolism alteration in follicular niche: The nexus among intermediary metabolism, mitochondrial function, and classic polycystic ovary syndrome. *Free Radic Biol Med* **2015,** *86*, 295-307.

2. Caicedo, D.; Diaz, O.; Devesa, P.; Devesa, J., Growth Hormone (GH) and Cardiovascular System. *Int J Mol Sci* **2018,** *19* (1).

3. Huang, D.; Cui, L.; Guo, P.; Xue, X.; Wu, Q.; Hussain, H. I.; Wang, X.; Yuan, Z., Nitric oxide mediates apoptosis and mitochondrial dysfunction and plays a role in growth hormone deficiency by nivalenol in GH3 cells. *Sci Rep* **2017,** *7* (1), 17079.

4. Rotterdam, E. A.-S. P. c. w. g., Revised 2003 consensus on diagnostic criteria and long-term health risks related to polycystic ovary syndrome (PCOS). *Hum Reprod* **2004,** *19* (1), 41-7.

5. Alpha Scientists in Reproductive, M.; Embryology, E. S. I. G. o., The Istanbul consensus workshop on embryo assessment: proceedings of an expert meeting. *Hum Reprod* **2011,** *26* (6), 1270-83.

6. Navot, D.; Bergh, P. A.; Laufer, N., Ovarian hyperstimulation syndrome in novel reproductive technologies: prevention and treatment. *Fertil Steril* **1992,** *58* (2), 249-61.

7. Wang, H.; Ruan, X.; Li, Y.; Cheng, J.; Mueck, A. O., Oxidative stress indicators in Chinese women with PCOS and correlation with features of metabolic syndrome and dependency on lipid patterns. *Arch Gynecol Obstet* **2019,** *300* (5), 1413-1421.

8. Zhang, R.; Liu, H.; Bai, H.; Zhang, Y.; Liu, Q.; Guan, L.; Fan, P., Oxidative stress status in Chinese women with different clinical phenotypes of polycystic ovary syndrome. *Clin Endocrinol (Oxf)* **2017,** *86* (1), 88-96.

9. Zhou, M.; Liu, X. H.; Liu, Q. Q.; Chen, M.; Bai, H.; Guan, L. B.; Fan, P., Lactonase Activity, Status, and Genetic Variations of Paraoxonase 1 in Women with Gestational Diabetes Mellitus. *J Diabetes Res* **2020,** *2020*, 3483427.

10. Verit, F. F.; Erel, O., Oxidative stress in nonobese women with polycystic ovary syndrome: correlations with endocrine and screening parameters. *Gynecol Obstet Invest* **2008,** *65* (4), 233-9.

**Research protocol: part 2**

**Budget**

Reagents and materials, $ 10,000.

Experiments, $ 5,000.

**Other support for the project**

None.

**Collaboration with other scientists or research institutions**

None.

**Curriculum Vitae of investigators**

Dr Wei Huang received her PhD in 1992 from West China Medical University, Chengdu Sichuan, China, and is currently full Professor of Obstetrics and Gynecology at the university. Her research focus is mainly polycystic ovary syndrome, endometriosis, female infertility, female human decidual stem cells and miRNA.

Dr Ping Fan graduated from the Department of Medicine at West China University of Medical Science in 1984. She specialized in biochemistry at the same university and got her master degree of medicine in 1991. In 1999, she went to Japan in the Department of Cardiology, Fukuoka University School of Medicine and gained her PhD in 2005. Her main proﬁciency and research interest is in lipid metabolism and the risk factors of cardiovascular diseases, and her research is particularly focused on PCOS and pregnancy-related diseases. She is currently an associate professor in West China Second University Hospital, Sichuan University.

Dr Yan Gong got her master degree of medicine at West China University of Medical Science in 2008. She is studying for PhD at the same university.Dr Shan Luo, Song Jin, Huili Zhu, Tang Deng and Yi Quan received their PhD from Sichuan University. They are currently associate professors or attending doctors in the Department of Obstetrics and Gynecology at the same university.

**Other research activities of the investigators**

The researches of the investigators focus on polycystic ovary syndrome, endometriosis, female infertility, female human decidual stem cells, lipid metabolism and the risk factors of cardiovascular diseases.

**Financing and insurance**

None.
